# Associations of Wearable-Measured Sleep and Physical Activity With Memory Performance in Older Adults: Cross-Sectional Study With Actigraphy and MRI

**DOI:** 10.2196/80584

**Published:** 2025-12-09

**Authors:** Geng-Hao Liu, Yueh-Hsiang Huang, Tzu-Chiao Yuan, Yueh-Peng Chen, Jung-Lung Hsu, Shwu-Hua Lee, Chih-Ming Lin, Cheng-Hong Toh, Ji-Tseng Fang, Shih-Wei Lin, Li-Pang Chuang, Ning-Hung Chen

**Affiliations:** 1 School of Traditional Chinese Medicine College of Medicine Chang Gung University Taoyuan Taiwan; 2 Graduate Institute of Traditional Chinese Medicine, School of Traditional Chinese Medicine College of Medicine Chang Gung University Taoyuan Taiwan; 3 Division of Acupuncture and Moxibustion Center for Traditional Chinese Medicine Linkou Chang Gung Memorial Hospital Taoyuan Taiwan; 4 Sleep Center Taoyuan Chang Gung Memorial Hospital Taoyuan Taiwan; 5 Division of Chinese Internal Medicine Center for Traditional Chinese Medicine Chang Gung Memorial Hospital Taipei Taiwan; 6 Taoying Fengze Chinese Medicine Clinic Fengze Chinese Medicine Clinic Taoyuan Taiwan; 7 Master of Science Degree Program in Innovation for Smart Medicine Chang Gung University Taoyuan Taiwan; 8 Center for Artificial Intelligence in Medicine Linkou Chang Gung Memorial Hospital Taoyuan Taiwan; 9 Division of Rheumatology, Allergy and Immunology Linkou Chang Gung Memorial Hospital Taoyuan Taiwan; 10 Department of Neurology New Taipei Municipal TuCheng Hospital (Built and Operated by Chang Gung Medical Foundation) New Taipei Taiwan; 11 Graduate Institute of Mind, Brain and Consciousness Taipei Medical University Taipei Taiwan; 12 Department of Psychiatry Linkou Chang Gung Memorial Hospital Taoyuan Taiwan; 13 College of Medicine Chang Gung University Taoyuan Taiwan; 14 Division of Internal Medicine Chang Gung Memorial Hospital Taipei Taiwan; 15 Department of Health Management Chang Gung Health and Culture Village Taoyuan Taiwan; 16 Department of Medical Imaging and Intervention Linkou Chang Gung Memorial Hospital Taoyuan Taiwan; 17 Kidney Research Center, Department of Nephrology Linkou Chang Gung Memorial Hospital Taoyuan Taiwan; 18 Department of Pulmonary and Critical Care Medicine Linkou Chang Gung Memorial Hospital Taoyuan Taiwan; 19 Department of Respiratory Therapy Linkou Chang Gung Memorial Hospital Taoyuan Taiwan

**Keywords:** CERAD-NB, cognitive aging, hippocampus, light physical activity, memory function, sleep fragmentation, wake after sleep onset, wearable devices

## Abstract

**Background:**

Cognitive decline is a common aspect of aging, and identifying modifiable lifestyle factors, such as physical activity and sleep, is crucial for promoting healthy brain aging. While both are individually linked to cognition, few studies have simultaneously assessed their independent and combined effects using objective wearable-based data, particularly in older Asian populations.

**Objective:**

This study aimed to examine the independent and interactive effects of wearable-assessed sleep and physical activity parameters on memory performance in healthy older adults. We also explored whether age and hippocampal volume moderated these associations.

**Methods:**

This prospective cross-sectional analysis included 88 cognitively healthy community-dwelling adults (≥60 years of age) from the *Integrating Systematic Data of Geriatric Medicine to Explore the Solution for Healthy Aging* cohort in Taiwan. Participants underwent 12-day wrist-worn actigraphy, brain magnetic resonance imaging, and neuropsychological assessments. Light-intensity physical activity (LPA) and wake after sleep onset (WASO) were selected based on age-adjusted partial correlations with Consortium to Establish a Registry for Alzheimer’s Disease Neuropsychological Battery memory scores. Multivariate regressions, age-stratified models (cutoff=72 years), and *PROCESS* moderation and mediation analyses were conducted, adjusting for age, education, daytime sleepiness, and hippocampal volume.

**Results:**

Partial correlation analyses adjusting for age showed that higher LPA (*r*=0.260; *P=*.02) and lower WASO (*r*=–0.251; *P=*.02) were significantly associated with better memory scores. Age significantly moderated both effects: LPA was beneficial beyond 73.8 years of age, and WASO was detrimental beyond 71.1 years of age. Multivariate regression models confirmed that both WASO (β=–.044; *P=*.04) and LPA (β=.042; *P=*.01) were significant predictors of memory. In subgroup analyses (age ≥72 years), both LPA (β=.054; *P*=.04) and WASO (β=–.111; *P*=.01) remained significant predictors. Moderated mediation analyses showed that WASO was associated with reduced LPA (β=–.325; *P*=.03), but the indirect effect on memory via LPA was not significant. Instead, WASO exerted a direct and age-moderated effect on memory performance. Hippocampal volume moderated both associations, supporting the brain reserve hypothesis.

**Conclusions:**

Our findings highlight WASO and LPA, as measured by wearable devices, as modifiable behavioral factors linked to memory function in older adults. The impact of these factors intensifies with advancing age and may be influenced by hippocampal reserve. Promoting daily light physical activity and maintaining sleep continuity may serve as accessible, age-tailored strategies for preserving cognitive health in aging populations.

**Trial Registration:**

ClinicalTrials.gov NCT04207502; https://classic.clinicaltrials.gov/ct2/show/NCT04207502

## Introduction

Cognitive decline is a well-recognized physiological aspect of aging. Even in the absence of dementia or mild cognitive impairment, older adults may experience subtle yet measurable decreases in memory performance and other cognitive domains [1]. These cognitive changes often occur alongside physical changes such as reductions in mobility, muscle strength, and endurance, which collectively affect quality of life and functional independence in late life. Among the most common behavioral alterations in aging populations are disrupted sleep patterns—such as changes in sleep timing and reduced sleep quality [2]—and reduced physical activity levels [3]. Growing evidence suggests that lifestyle factors play a critical role in both physical and cognitive health in older adults [4-6]. Given the lack of well-established pharmacological treatments for early-stage cognitive decline, modifiable lifestyle behaviors such as sleep and physical activity are increasingly recognized as vital contributors to cognitive reserve and healthy brain aging.

Many studies have investigated the association between self-reported sleep quality and cognitive function, although the findings have been inconsistent. Some reports have linked longer sleep latency and longer or shorter total sleep duration with poorer cognitive outcomes [7-10], including U-shaped relationships between sleep duration and cognitive performance. Studies using objective sleep measures have further identified associations between poor sleep quality—characterized by increased wake after sleep onset (WASO), prolonged sleep latency, and reduced sleep efficiency—and higher risk of cognitive impairment [11,12].

Physical activity is another important modifiable factor associated with cognitive aging. Observational studies have shown that higher levels of physical activity are linked to better cognitive function in older adults [13,14], whereas sedentary behavior (SB) is associated with poorer outcomes [15]. Interventional trials have also demonstrated cognitive benefits from regular physical exercise [16,17]. Mechanistically, physical activity may promote brain health through the release of muscle-derived myokines, which support neuroplasticity and anti-inflammatory processes [18]. Additionally, sarcopenia has been associated with cognitive decline, highlighting the potential interplay between muscle and brain health [19].

Although a growing body of literature has explored the impact of sleep or physical activity on cognitive functioning, studies that simultaneously examine both factors using objective, device-based methods remain limited, particularly among older adults in Asian populations. Furthermore, little is known about how these associations vary across different age thresholds in older people, despite evidence that the aging process may alter the relative influence of these lifestyle behaviors. In this study, we aimed to investigate the associations between sleep characteristics, physical activity levels, and memory function in healthy, community-dwelling older adults in Taiwan, using actigraphy-based monitoring and age-stratified as well as moderation analyses to explore potential age-related differences.

## Methods

### Study Population

This study was part of a prospective observational cohort established to create a comprehensive geriatric database for community-dwelling older adults in Taiwan without major physical or psychological disabilities. Community-dwelling adults aged 60 years and older were invited to participate in the *Integrating Systematic Data of Geriatric Medicine to Explore the Solution for Healthy Aging (ISDHA)* study between September 2019 and October 2020 during annual routine health examinations at Chang Gung Memorial Hospital. Participants were residents of either the Chang Gung Health and Culture Village in Taoyuan or the Songshan District in Taipei, Taiwan. The inclusion criteria were as follows: (1) aged ≥60 years, (2) had visited Chang Gung Memorial Hospital at least once within the past year, and (3) had resided in Taiwan for more than 180 days within the year prior to enrollment. Exclusion criteria included (1) clinical evidence of major organ dysfunction; (2) a history of severe autoimmune disease; (3) ongoing cancer treatment at the time of recruitment; (4) antibiotic use within 1 month prior to enrollment; (5) cognitive impairment, defined as an Ascertain Dementia 8 (AD8) questionnaire score of ≥2 or Mini-Mental State Examination (MMSE) score of ≤26; (6) current follow-up at a cognitive disorder outpatient clinic; (7) depressive symptoms, defined as a Geriatric Depression Scale (GDS) score of ≥5; (8) a prior diagnosis of dementia or major depressive disorder; (9) severe hearing, visual, or cognitive impairment, preventing meaningful participation in interviews; or (10) physical frailty precluding the ability to stand or walk independently.

For this analysis, only participants who completed brain magnetic resonance imaging (MRI) and objective assessments of sleep quality and physical activity using actigraphy were included.

### Demographic and Clinical Data Collection

Demographic information, including age, educational attainment, smoking status, and habitual exercise, was obtained through structured interviews. Medical history, such as the presence of hypertension, hyperlipidemia, diabetes mellitus, insomnia, obstructive sleep apnea, peptic ulcer disease, and anxiety, was collected from both electronic medical records and participant self-reports. Vital signs, including systolic and diastolic blood pressure and resting pulse rate, were measured using an automated sphygmomanometer. Body height and weight were measured using standardized procedures, and BMI was calculated as weight in kilograms divided by the square of height in meters (kg/m^2^).

### Blood Sample Collection and Laboratory Tests

Fasting venous blood samples were collected from all participants following an overnight fast of 8-12 hours. Laboratory tests included a complete blood count, liver and renal function panels, metabolic markers, serum vitamin D and albumin levels, as well as proinflammatory cytokines. All analyses were performed in the central laboratory of Chang Gung Memorial Hospital using standardized protocols.

### Body Composition Assessments

Body composition was assessed using bioelectrical impedance analysis, a noninvasive and reliable method based on the electrical conductivity properties of different tissues [20]. Measurements were performed using a multifrequency segmental body composition analyzer (Tanita MC-780 MA; Tanita Corp), following the manufacturer’s standard operating procedures. The parameters obtained included fat mass (kg), fat-free mass (kg), percentage of body fat, visceral fat rating, muscle mass (kg), bone mass (kg), total body water (TBW; kg), intracellular water (kg), extracellular water (ECW; kg), and basal metabolic rate (kcal).

### Cognitive Function Assessments

Global cognitive status was assessed using the MMSE [21] and the AD8 questionnaire [22]. To specifically evaluate memory function, the Consortium to Establish a Registry for Alzheimer’s Disease Neuropsychological Battery (CERAD-NB) was administered following standardized procedures [23]. The CERAD-NB is a widely validated tool for assessing cognitive performance in normal aging and Alzheimer disease [24] and includes several subtests: Verbal Fluency, the Modified Boston Naming Test, MMSE, Word List Memory, and Constructional Praxis [25]. The memory total score of the CERAD-NB was calculated by summing the scores from the 3 trials of the Word List Learning test, Delayed Recall, and Recognition subtests, with a maximum total score of 50 [25].

### Subjective Sleep Assessments

Subjective sleep quality and daytime sleepiness were assessed using 2 standardized self-report questionnaires. The Pittsburgh Sleep Quality Index (PSQI) was used to evaluate overall sleep quality over the past month, while the Epworth Sleepiness Scale (ESS) assessed the general level of daytime sleepiness [26]. Both instruments have been widely validated in older adult populations and are commonly used in aging and sleep research.

### Actigraphy-Based Monitoring of Physical Activity and Sleep

Objective monitoring of physical activity and sleep was performed using a wrist-worn accelerometer (Actiwatch 2; Philips Respironics), which incorporates a piezoelectric accelerometer to detect movement and a photodiode sensor to measure ambient light (illuminance in lux). The device recorded activity as counts per minute and light exposure at 60-second epochs. Participants were instructed to wear the device on the nondominant wrist for 14 consecutive days. Data from the first and last days were excluded from analysis to avoid partial-day bias. Sleep and wake periods were scored by a trained sleep specialist using Philips Actiware software (version 6.0.9) in conjunction with participants’ sleep diaries. Epochs with activity counts below the wake threshold were classified as sleep, while those above the threshold were classified as wake. Circadian rhythm parameters were derived using nonparametric analysis. M10 and L5 were defined as the mean activity counts per minute during the most active 10 consecutive hours and the least active 5 consecutive hours of the day, respectively, reflecting rest-activity patterns [27].

Actigraphy recordings were considered valid if participants wore the device for at least 7 valid days (≥10 hours of daytime wear per day) without prolonged removal. Participants with fewer than 7 valid days (n=1) or who did not complete the actigraphy assessment (n=7) were excluded from the final analysis. The average number of valid actigraphy days before excluding the first and last partial days was 13.6 (SD 1.3; range 9-14) days. After trimming these 2 boundary days to avoid partial-day bias, a continuous 12-day dataset was used for the final analysis. Participants wore the Actiwatch 2 for an average of 1412 (SD 12.5) minutes per day (~23.5 hours), with all valid recordings exceeding 870 minutes per day (~14.5 hours), indicating high adherence and minimal device removal during monitoring. Most participants (84/88, 95.5%) provided 13-14 days of valid recordings, demonstrating excellent compliance and data completeness.

Physical activity parameters were categorized using validated counts-per-minute thresholds obtained from Actiwatch 2 actigraphy data [28]. SB was defined as activity of <145 counts/minute (corresponding to <1.5 metabolic equivalents of task [METs]), light-intensity physical activity (LPA) as 145-274 counts/minute (~1.5-3 METs), moderate-intensity physical activity (MPA) as 274-597 counts/minute (~3-6 METs), and vigorous-intensity physical activity (VPA) as >597 counts/minute (>6 METs). Wake activity time was calculated as the total time spent in any activity category (SB, LPA, MPA, or VPA) during waking hours. Total activity counts during the wake period were also recorded as an indicator of overall activity level.

Light exposure during the wake period was evaluated using the following measures: wake light illuminance, defined as the average light intensity (lux); wake light time, representing the total duration (minutes) exposed to light; and wake light exposure, calculated as the cumulative product of light intensity and time (lux × minutes).

Sleep-related parameters were also derived from the actigraphy data. These included time in bed (TIB), defined as the duration between bedtime and final wake time; total sleep time (TST), referring to the total number of minutes scored as sleep; sleep onset latency, defined as the time from attempting to sleep to sleep onset; sleep efficiency, calculated as the percentage of time asleep while in bed; and WASO, representing the total number of minutes awake between sleep onset and the final awakening. In addition, light exposure during the sleep period was evaluated, including sleep light illuminance (average light intensity), sleep light time (duration of light exposure during sleep), sleep light exposure (lux × minutes), and sleep total activity (cumulative movement counts during sleep).

Summary statistics of subjective questionnaires and actigraphy-derived parameters, including sleep, physical activity, and light exposure, are presented in Textbox 1.

**Clinical assessments included subjective questionnaires (Pittsburgh Sleep Quality Index [PSQI] and Epworth Sleepiness Scale [ESS]) and actigraphy-derived parameters for sleep, physical activity, and light exposure. The actigraphy-derived parameters were obtained from 12 consecutive days of wrist-worn recordings.**

**Subjective assessments**

- Sleep parameters include (1) PSQI (past 1 month) and (2) ESS (past 1 month).

**Objective assessments**

- Actigraphy-derived sleep parameters include (1) time in bed (min), (2) total sleep time (min), (3) onset latency (min), (4) sleep efficiency (%), and (5) wake after sleep onset (min).
- Actigraphy-derived physical activity parameters include (1) M10 (counts/min; indicates the mean activity during the most active 10 consecutive hours), (2) L5 (counts/min; indicates the mean activity during the least active 5 consecutive hours), (3) awaking total activity (counts), (4) sleep total activity (counts), and (5) physical activity time (min), which is further categorized into (i) sedentary behavior time (min), (ii) light-intensity physical activity time (min), (iii) moderate-intensity physical activity time (min), and (iv) vigorous-intensity physical activity time (min).
- Actigraphy-derived light exposure parameters include (1) wake light exposure (lux-min) and (2) sleep light exposure (lux-min).

### Brain MRI Acquisition and Analysis

Brain MRI was performed using 3-Tesla scanners (Discovery MR750w and Discovery MR750; GE Healthcare). For each participant, 3 imaging sequences were acquired: a 3D T1-weighted anatomical scan, a 3D T2 fluid-attenuated inversion recovery scan, and diffusion tensor imaging. Gray matter and white matter volumes were estimated from the high-resolution T1-weighted images using the Computational Anatomy Toolbox (version 12.7), implemented within the Statistical Parametric Mapping software (version 12; Wellcome Trust Centre for Neuroimaging).

### Statistical Analysis

All statistical analyses were performed using SPSS Statistics software (version 25.0; IBM Corp). Descriptive statistics were used to summarize participant characteristics, including age, education level, sleep and physical activity parameters, and cognitive performance. The normality of continuous variables was assessed using the Kolmogorov-Smirnov test. As most variables did not meet the assumption of normality, nonparametric methods were applied, and between-group comparisons were conducted using the Mann-Whitney *U* test.

To examine the associations between actigraphy-derived sleep and physical activity variables and memory performance, partial correlation analyses adjusted for age were conducted. Based on these results, WASO (*r*=–0.251; *P=*.02) and LPA time (*r*=0.26; *P=*.02) were identified as candidate modifiable lifestyle factors and selected for further analysis. Subsequently, multivariate linear regression analyses were performed to evaluate the independent contributions of WASO and LPA time to CERAD-NB memory scores, adjusting for relevant covariates, including age, education level, ESS score, and mean hippocampal volume. To investigate whether the effects of lifestyle factors on memory performance were moderated by age, moderation analyses were conducted using the *PROCESS* macro (version 4.2) for SPSS, developed by Andrew F Hayes. Model 1 was used to test interaction effects between age and each lifestyle factor (eg, age × WASO), and the Johnson-Neyman technique was applied to identify the age threshold at which the interaction became statistically significant. Based on these results, a cutoff age of ≥72 years was established and used for subsequent age-stratified regression analyses.

**Figure 1:**
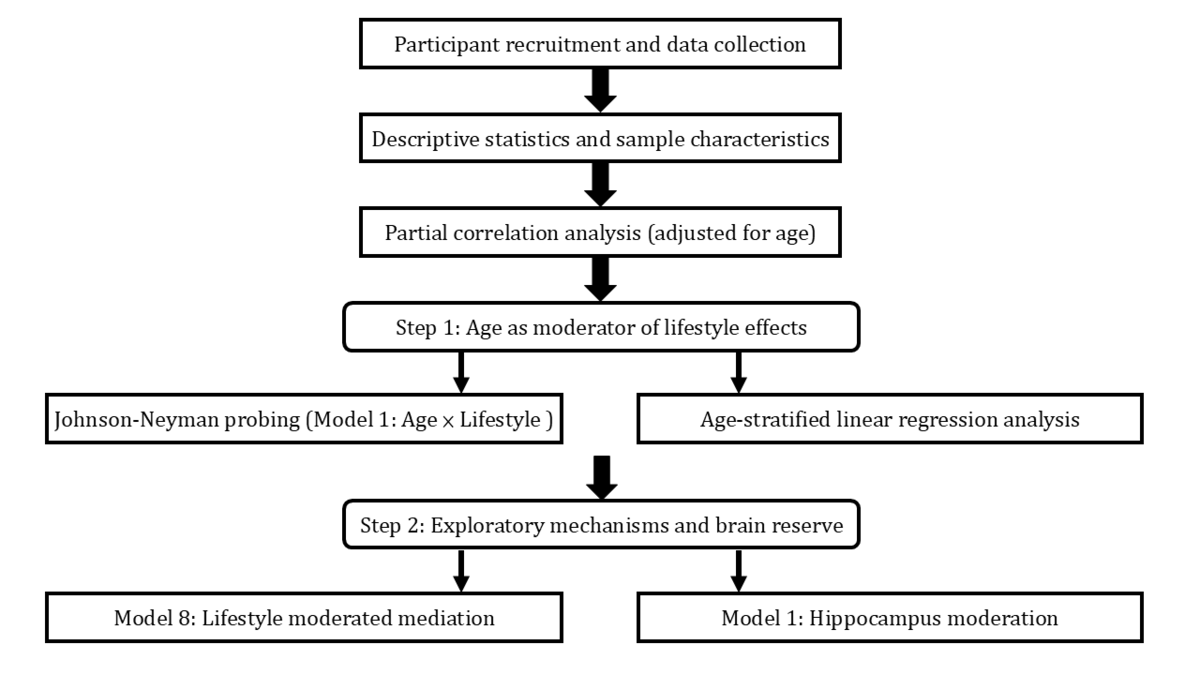
Analysis flowchart of the study design.

Finally, exploratory analyses were conducted to examine potential mechanisms linking sleep and physical activity to memory performance. This included moderated mediation analyses using Model 8 of the *PROCESS* macro to assess indirect effects and additional moderation analyses (Model 1) evaluating the role of hippocampal volume as a potential moderator, in alignment with the brain reserve hypothesis. All statistical tests were 2-tailed, with *P* values of <.05 considered statistically significant. For moderation and mediation models, bias-corrected bootstrap 95% CIs based on 5000 resamples were used to assess the robustness of indirect and interaction effects. The overall analytic framework is illustrated in Figure 1.

This diagram illustrates the sequential analytic framework used to investigate the associations between lifestyle factors and memory performance in older adults. After participant recruitment and descriptive analysis, partial correlation analysis (adjusted for age) was conducted to identify candidate actigraphy-derived lifestyle parameters. Step 1 examined whether age moderated the relationship between these lifestyle variables and memory performance, using Johnson-Neyman probing (*PROCESS* Model 1) and age-stratified linear regression. Step 2 further explored potential mechanisms and the brain reserve hypothesis through lifestyle-moderated mediation analysis (Model 8) and mean hippocampal volume moderation (Model 1).

### Ethical Considerations

The study was approved by the Institutional Review Board of Chang Gung Medical Foundation, Taiwan (approval number 201900702A3). Written informed consent was obtained from all participants. To ensure privacy and confidentiality, all data were deidentified using unique study codes, with no personally identifiable information stored with research data. Participants received NT $100 (US $3.10) for nutritional supplementation and NT $200 (US $6.20) for transportation for each visit involving additional blood sampling or questionnaire-based assessments.

## Results

### Participants’ Characteristics

**Figure 2:**
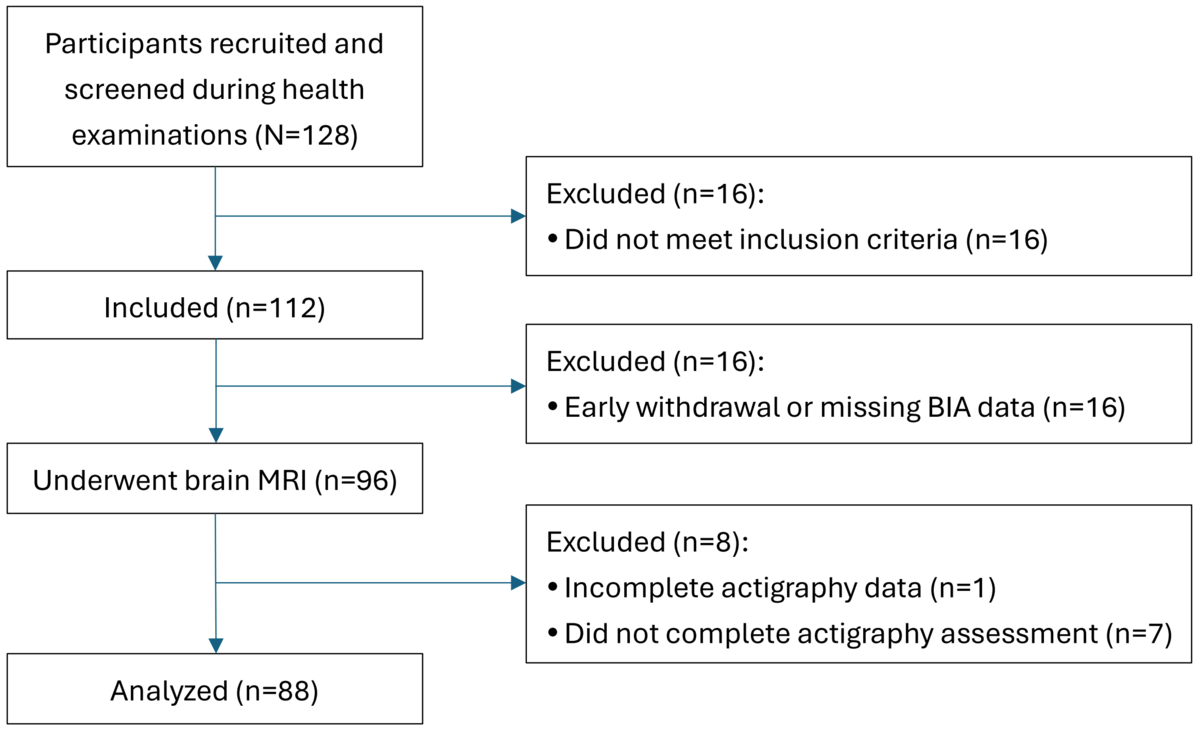
Flow diagram of the inclusion process. BIA: bioelectrical impedance analysis; MRI: magnetic resonance imaging.

Of the 128 individuals initially enrolled in the study, 112 met the inclusion criteria. Participants who did not complete the body composition assessment (bioelectrical impedance analysis), brain MRI, or valid actigraphy recordings were excluded. After these exclusions, a total of 88 participants were included in the final analysis (flow diagram in Figure 2). Their demographic characteristics, cognitive performance, subjective sleep assessments, and actigraphy-derived sleep and physical activity parameters are summarized in the total column of Table 1.

The mean age of participants was 73.5 (SD 7) years, and 55.7% (49/88) were female. Educational attainment was distributed as follows: 8% (7/88) had completed primary school, 23.9% (21/88) had completed junior or senior high school (including vocational programs), 52.3% (46/88) had attended college or university, and 15.9% (14/88) held a graduate degree. The mean BMI was 23.7 (SD 3.4) kg/m^2^, and the average systolic and diastolic blood pressures were 138.4 (SD 17) mm Hg and 75 (SD 9.6) mm Hg, respectively. Regarding habitual exercise, 75% (66/88) of participants reported engaging in regular physical activity exceeding 150 minutes per week, 19.3% (17/88) exercised less than 150 minutes per week, and only 5.7% (5/88) reported no regular exercise habit.

The average CERAD-NB memory score was 37 (SD 5.7). In terms of subjective sleep assessments, the mean scores were 6.4 (SD 3.8) for the PSQI and 3.7 (SD 3.7) for the ESS.

Actigraphy-derived sleep parameters revealed an average TIB of 468 (SD 81.2) minutes and TST of 391.5 (SD 80.7) minutes. The mean sleep onset latency was 11.6 (SD 13.4) minutes, sleep efficiency was 83.6% (SD 8.2%), and WASO averaged 56.2 (SD 25.6) minutes. In terms of physical activity, participants showed an average M10 activity of 267 (SD 97.2) counts/minute and L5 activity of 21.7 (SD 15.6) counts/minute. Daily activity distributions included a mean LPA time of 141.5 (SD 33.2) minutes, MPA of 236.2 (SD 67.6) minutes, and VPA of 141.3 (SD 88.5) minutes. Average wake light exposure was 136576.4 (SD 129264.3) lux-minute, and sleep light exposure was 497.9 (SD 1103.9) lux-minute. A comprehensive summary of subjective and actigraphy-derived sleep and activity parameters is provided in Textbox 1.

**Table 1:** Characteristics of 88 participants analyzed in the *Integrating Systematic Data of Geriatric Medicine to Explore the Solution for Healthy Aging* study.

Demographics				Total (n=88)		Age						*P* value			
						<72 years (n=44)		≥72 years (n=44)							
Age (years), mean (SD)				73.5 (7)		67.9 (2.4)		79.1 (5.3)			<.001				
Sex (female), n (%)				49 (55.7)		25 (56.8)		24 (54.5)			.83				
Systolic blood pressure (mm Hg), mean (SD)				138.4 (17)		135 (14.7)		141.7 (18.5)			.08				
Diastolic blood pressure (mm Hg), mean (SD)				75 (9.6)		74.9 (10.3)		75.1 (8.8)			.95				
BMI (kg/m^2^), mean (SD)				23.7 (3.4)		23.2 (2.8)		24.2 (3.8)			.33				
**Education, n (%)**												.34			
	Primary school		7 (8)		3 (6.8)		4 (9.1)								
	Junior or senior high (including vocational)		21 (23.9)		9 (20.5)		12 (27.3)								
	University or college		46 (52.3)		24 (54.5)		22 (50)								
	Graduate school		14 (15.9)		8 (18.2)		6 (13.6)								
**Exercise (in 1 week), n (%)**												.52			
	No exercise habit		5 (5.7)		1 (2.3)		4 (9.1)								
	<150 minutes		17 (19.3)		9 (20.5)		8 (18.2)								
	≥150 minutes		66 (75)		34 (77.3)		32 (72.7)								
**Questionnaires, mean (SD)**															
	CERAD-NB^a^ memory score		37 (5.7)		38.7 (4.2)		35.4 (6.5)			.02					
	Pittsburgh Sleep Quality Index		6.4 (3.8)		6.5 (3.9)		6.3 (3.7)			.90					
	Epworth Sleepiness Scale		3.7 (3.7)		3.7 (3.7)		3.8 (3.7)			.96					
**Actigraphy-derived sleep parameters, mean (SD)**															
	Time in bed (min)		468 (81.2)		444.3 (67.6)		491.8 (87.2)			.006					
	Total sleep time (min)		391.5 (80.7)		370.5 (73.1)		412.5 (83.2)			.02					
	Onset latency (min)		11.6 (13.4)		11.2 (17)		12 (8.6)			.17					
	Sleep efficiency (%)		83.6 (8.2)		83.3 (10.5)		83.9 (5.2)			.62					
	Wake after sleep onset (min)		56.2 (25.6)		52.9 (27.3)		59.4 (23.8)			.12					
**Actigraphy-derived physical activity parameters, mean (SD)**															
	M10^b^ (counts/min)		267 (97.2)		296.9 (99.9)		237.1 (85.4)			.006					
	L5^c^ (counts/min)		21.7 (15.6)		21.4 (20)		22.1 (9.8)			.06					
	Awaking total activity (counts)		280508.1 (107005.9)		291065.6 (107660.9)		230846.9 (80836.7)			.007					
	Sleep total activity (counts)		9119.8 (4950.1)		7796.7 (5378.5)		9549.3 (3944.9)			.005					
	**Physical activity time (min)**		972.6 (81.9)		996.7 (66.2)		948.5 (89.4)			.01					
		SB^d^ time (min)	453.7 (117.5)		441.8 (121.7)				465.5 (113.2)		.34				
		LPA^e^ time (min)	141.5 (33.2)		140.1 (30.2)				143 (36.2)		.55				
		MPA^f^ time (min)	236.2 (67.6)		240.5 (58.2)				231.9 (76.2)		.81				
		VPA^g^ time (min)	141.3 (88.5)		174.4 (99.1)				108.2 (61.6)		<.001				
**Actigraphy-derived light exposure parameters (lux-min), mean (SD)**															
	Wake light exposure		136576.4 (129264.3)		175062.9 (149387.8)		98089.9 (91973.2)			<.001					
	Sleep light exposure		497.9 (1103.9)		247.7 (362.3)		748 (1485.4)			.32					

^a^CERAD-NB: Consortium to Establish a Registry for Alzheimer’s Disease Neuropsychological Battery.

^b^M10 indicates the mean activity (counts/min) during the most active 10 consecutive hours.

^c^L5 indicates the mean activity (counts/min) during the least active 5 consecutive hours.

^d^SB: Sedentary behavior.

^e^LPA: Light-intensity physical activity.

^f^MPA: moderate-intensity physical activity.

^g^VPA: vigorous-intensity physical activity.

### Correlation Between Sleep, Physical Activity, and Memory Performance

Partial correlation analyses adjusted for age were conducted to examine the associations between actigraphy-derived parameters and memory performance, as measured by the CERAD-NB memory score (Table 2).

Among sleep-related variables, WASO showed a significant negative correlation with memory performance (*r*=–0.251; *P=*.02), indicating that greater sleep fragmentation was associated with lower memory scores. Other sleep parameters, such as TIB (*r*=–0.199; *P=*.07), sleep efficiency (*r*=0.152; *P=*.16), and onset latency (*r*=–0.119; *P=*.28), did not reach statistical significance.

**Table 2:** Partial correlations between actigraphy parameters and Consortium to Establish a Registry for Alzheimer’s Disease Neuropsychological Battery memory score.

Actigraphy variables					Values (adjusted for age)			
					*r*			*P* value
**Sleep**								
	Time in bed (min)			–0.199			.07	
	Total sleep time (min)			–0.082			.45	
	Onset latency (min)			–0.119			.28	
	Sleep efficiency (%)			0.152			.16	
	Wake after sleep onset (min)			–0.251			.02	
**Physical activity**								
	M10^a^ (counts/min)			0.216			.046	
	L5^b^ (counts/min)			–0.098			.37	
	Awaking total activity (counts)			0.166			.13	
	Sleep total activity (counts)			–0.160			.14	
	**Physical activity time (min)**			0.196			.07	
		SB^c^ time (min)	–0.132			.23		
		LPA^d^ time (min)	0.260			.02		
		MPA^e^ time (min)	0.170			.12		
		VPA^f^ time (min)	0.120			.27		
**Light exposure**								
	Wake light exposure (lux-min)			–0.144			.19	
	Sleep light exposure (lux-min)			–0.167			.12	

^a^M10 indicates the mean activity (counts/min) during the most active 10 consecutive hours.

^b^L5 indicates the mean activity (counts/min) during the least active 5 consecutive hours.

^c^SB: sedentary behavior.

^d^LPA: light-intensity physical activity.

^e^MPA: moderate-intensity physical activity.

^f^VPA: vigorous-intensity physical activity.

Regarding physical activity, several metrics showed modest positive correlations with memory. Notably, LPA time was significantly associated with better memory scores (*r*=0.260; *P=*.02). Additionally, M10—the mean activity (counts/min) during the most active 10 consecutive hours—was also significantly correlated with memory scores (*r*=0.216; *P=*.046). However, LPA was prioritized for subsequent multivariate and moderation analyses because it represents a clearly defined, low-intensity, and modifiable behavioral category, whereas M10 reflects a composite index of total daytime movement combining multiple activity intensities. Thus, LPA provided a more interpretable and intervention-relevant indicator for modeling behavioral effects on memory. Total physical activity time (*r*=0.196; *P=*.07) and MPA (*r*=0.170; *P=*.12) showed nonsignificant trends in the expected direction. No significant associations were observed for sedentary time, VPA, or nighttime sleep activity.

Light exposure variables, including wake light exposure and sleep light exposure, also did not show significant correlations with memory performance (*P*>.10). Taken together, WASO and LPA time emerged as the 2 behavioral parameters showing statistically significant and physiologically meaningful associations with memory and were therefore selected for subsequent regression and moderation models to examine their independent and age-dependent effects on cognitive performance.

### Age as a Moderator of the Association Between Lifestyle Factors and Memory

**Figure 3:**
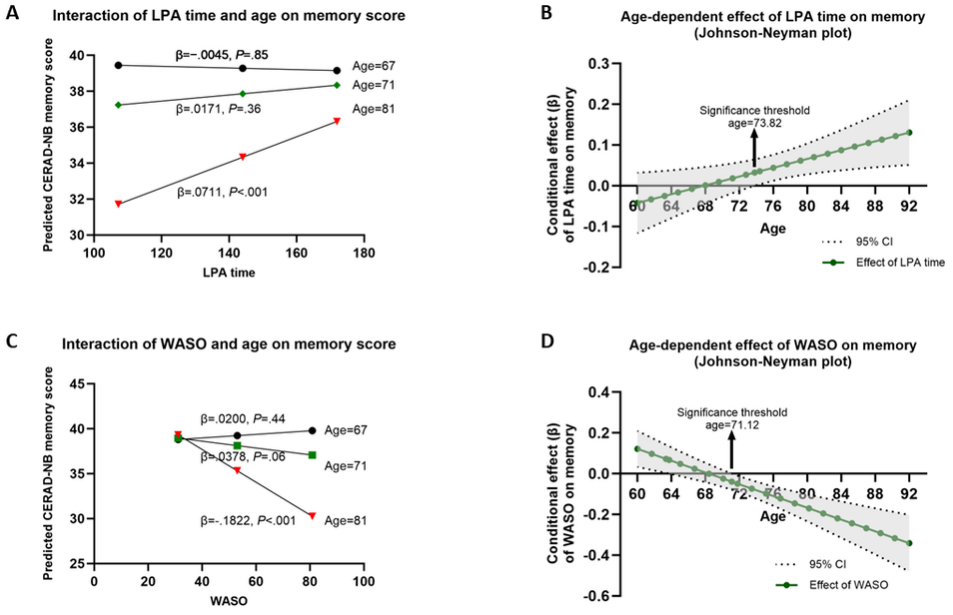
Interaction effects of age with light-intensity physical activity (LPA) time and wake after sleep onset (WASO) on Consortium to Establish a Registry for Alzheimer’s Disease Neuropsychological Battery (CERAD-NB) memory performance. (A) Interaction between LPA time and age on CERAD-NB memory scores. (B) Johnson-Neyman plot showing the age-dependent significance threshold for the association between LPA time and memory. (C) Interaction between WASO and age on CERAD-NB memory scores. (D) Johnson-Neyman plot showing the age-dependent significance threshold for the association between WASO and memory.

To determine whether age moderated the relationship between modifiable lifestyle factors and memory performance, moderation analyses were conducted using *PROCESS* Model 1 with the Johnson-Neyman technique. As illustrated in Figure 3, age significantly moderated both associations of LPA time and WASO with CERAD-NB memory scores. In contrast, no significant moderating effects of age were observed for MPA, VPA, or combined moderate to vigorous physical activity (MVPA) on memory performance (Figure S1 in Multimedia Appendix 1).

Figure 3A illustrates the interaction between LPA time and age on CERAD-NB memory scores. While no significant association was found in younger adults (eg, aged 67 or 71 years), higher LPA time was significantly associated with better memory performance at older ages (eg, 81 years; β=.0711; *P*<.001). Figure 3B shows the Johnson-Neyman plot, indicating that the association between LPA time and memory became statistically significant at age 73.82 years onward.

Figure 3C depicts the interaction between WASO and age. While WASO was not significantly associated with memory among younger individuals, it showed a significant negative relationship at older ages (eg, 81 years; β=–.1822; *P*<.001). The Johnson-Neyman plot in Figure 3D indicates a significance threshold at age 71.12 years, after which increased WASO was associated with worse memory performance.

All models were tested using the *PROCESS* macro Model 1 (moderation analysis), examining the interaction between age and each lifestyle factor.

For LPA time, the conditional effect on memory became statistically significant at age 73.82 years, above which greater LPA time was associated with higher memory scores. Conversely, for WASO, the association with memory became significantly negative starting from age 71.12 years, indicating that sleep fragmentation had a more detrimental impact on memory among older participants. Based on these results, we stratified participants into 2 groups using an age cutoff of 72 years for subsequent subgroup analyses.

Compared to younger participants, older adults (aged ≥72 years), as shown in the age-stratified columns of Table 1, had significantly lower CERAD-NB memory scores (35.4, SD 6.5 minutes vs 38.7, SD 4.2; *P=*.02), longer TIB (491.8, SD 87.2 minutes vs 444.3, SD 67.6 minutes; *P=*.006), and greater TST (412.5, SD 83.2 minutes vs 370.5, SD 73.1 minutes; *P=*.02). However, no significant age differences were observed in sleep onset latency, sleep efficiency, or WASO.

In terms of physical activity, participants aged ≥72 years had significantly lower M10 activity (237.1, SD 85.4 counts/min vs 296.9, SD 99.9 counts/min; *P=*.006), shorter VPA time (108.2, SD 61.6 minutes vs 174.4, SD 99.1 minutes; *P*<.001), and reduced awake total activity and sleep total activity counts (*P*<.01). They also exhibited significantly lower wake light exposure (*P*<.001).

Further comparisons in Table S1 in Multimedia Appendix 1 revealed significant age-related physiological and structural differences. Older participants had slower gait speed, lower estimated glomerular filtration rate, and smaller hippocampal volume, including both right and left hippocampi (*P*<.001). Additionally, significant age-related differences were observed in visceral fat rating, ECW and TBW ratio, and regional brain structure volumes (eg, anterior cingulate cortex), consistent with expected patterns of biological aging. Moreover, among the inflammatory markers assessed, only interleukin-8 showed a significant increase with age (*P*=.02), whereas other cytokines (interleukin-1β, interleukin-6, interleukin-10, tumor necrosis factor-alpha) did not differ significantly between groups, suggesting mild low-grade inflammation in this otherwise healthy cohort.

Together, these findings support the use of age-stratified analyses to further explore how sleep and activity factors impact memory differently across the aging continuum.

### Multivariate Models and Brain-Based Mechanisms

To assess the independent contributions of LPA time and WASO to memory performance, multivariate linear regression analyses were conducted, adjusting for age, education level, ESS scores, and mean hippocampal volume. As shown in Table 3, both LPA time (β=.042; *P*=.01) and WASO (β=–.044; *P*=.04) remained significant predictors of CERAD-NB memory scores in the overall sample. Increasing age was independently associated with lower memory scores (β=–.318; *P*=.003), whereas education level, ESS, and hippocampal volume did not show significant contributions in the total model. Among participants aged ≥72 years, the effects of LPA time (β=.054; *P*=.04) and WASO (β=–.111; *P*=.01) on memory remained statistically significant, suggesting that these modifiable lifestyle factors exert a stronger influence in later age.

**Table 3:** Multivariate linear regression models examining associations of light-intensity physical activity (LPA) time and wake after sleep onset (WASO) with Consortium to Establish a Registry for Alzheimer’s Disease Neuropsychological Battery memory scores. Two adjusted models are presented: the left column reports results for the full sample, and the right column reports results for participants aged ≥72 years. Both overall models were statistically significant (*P*<.001). All models were adjusted for age, education level, Epworth Sleepiness Scale score, and mean hippocampal volume. Regression coefficients (β) and corresponding *P* values are reported.

Factors	Models (total)		Models (age ≥72 years)	
	β	*P* value	β	*P* value
Age	–.318	.003	–.268	.15
Education	1.144	.09	.886	.39
Epworth Sleepiness Scale	–.207	.17	–.257	.25
Mean hippocampal volume	.054	.98	6.053	.09
LPA time	.042	.01	.054	.04
WASO	–.044	.04	–.111	.01

**Figure 4:**
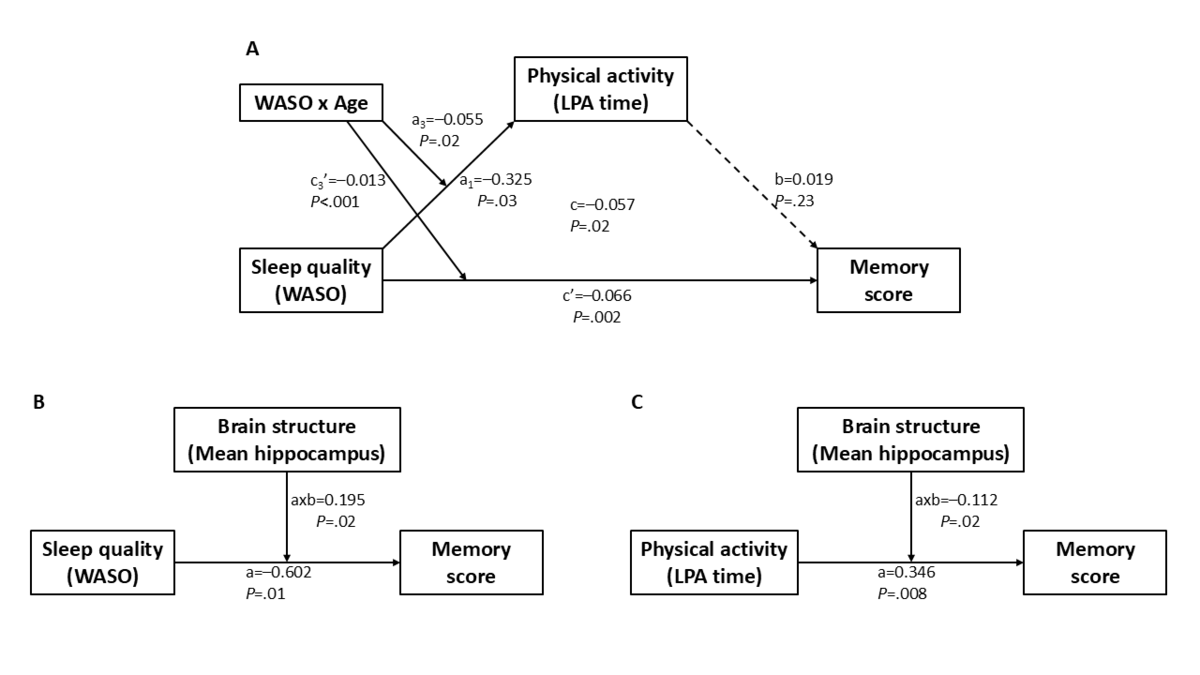
Exploratory models examining potential mechanisms linking sleep fragmentation, physical activity, and memory performance in older adults. (A) Moderated mediation model examining whether the association between wake after sleep onset (WASO) and memory performance is mediated by light-intensity physical activity (LPA) time and moderated by age. (B) Moderation model testing whether mean hippocampal volume moderates the association between WASO and memory performance. (C) Moderation model testing whether mean hippocampal volume moderates the association between LPA time and memory performance.

To further explore the potential mechanisms linking sleep fragmentation, physical activity, and memory decline, 2 exploratory models were tested using the *PROCESS* macro (Figure 4A). A moderated mediation model (Model 8) showed that higher WASO was associated with lower LPA time (a_1_=–0.325; *P*=.03), and this relationship was significantly moderated by age (a_3_=–0.055; *P*=.02), suggesting that sleep disruption more strongly reduced activity levels in older adults. However, LPA itself was not a significant predictor of memory performance (b=0.019; *P*=.23), and the indirect effect of WASO on memory via LPA was not statistically significant. Instead, WASO exerted a direct effect on memory performance (c’=–0.066; *P*=.002) that was also moderated by age (c_3_’=–0.013; *P*<.001), supporting an age-dependent vulnerability to sleep fragmentation on cognitive function.

In Figure 4A, a moderated mediation model (*PROCESS* Model 8) was used to examine whether the relationship between sleep fragmentation (measured by WASO) and memory performance was mediated by LPA time and whether age moderated these behavioral pathways. Higher WASO was associated with reduced LPA (a_1_=–0.325; *P*=.03), and this effect was moderated by age (a_3_=–0.055; *P*=.02), indicating a stronger negative effect of sleep disruption on physical activity in older individuals. However, LPA was not a significant predictor of memory performance (b=0.019; *P*=.23), and the indirect path from WASO to memory via LPA was not statistically significant. In contrast, the direct effect of WASO on memory remained significant after accounting for LPA (c’=–0.066; *P*=.002), and this effect was also moderated by age (c_3_’=–0.013; *P*<.001), indicating that the negative impact of WASO on memory increased with advancing age.

In addition, moderation analyses (*PROCESS* Model 1) were used to test the brain reserve hypothesis by examining hippocampal volume as a moderator of the LPA-memory and WASO-memory associations (Figures 4B and 4C). For WASO, hippocampal volume significantly moderated the association with memory (interaction effect: a×b=0.195; *P*=.02), suggesting that individuals with greater hippocampal volume were less affected by sleep fragmentation. Similarly, for LPA, the moderation effect was significant (a×b=–0.112; *P*=.02), indicating that low hippocampal reserve amplified the cognitive impact of reduced physical activity.

These findings provide evidence for both behavioral (activity-mediated) and structural (hippocampal-modulated) pathways linking modifiable lifestyle factors to memory outcomes in older adults.

In Figure 4B, moderation models (*PROCESS* Model 1) examined whether mean hippocampal volume moderated the effects of WASO and LPA on memory, consistent with the brain reserve hypothesis. These models showed that mean hippocampal volume significantly moderated the relationships between both WASO (a=–0.602; *P*=.01; a×b=0.195; *P*=.02) and LPA (a=0.346; *P*=.008; a×b=–0.112; *P*=.02) with memory scores. These findings support the idea that greater structural brain reserve may buffer the negative impact of sleep disruption and low activity on cognitive outcomes in aging.

These findings underscore WASO as a direct and age-sensitive predictor of memory performance, with its negative impact particularly evident in older individuals and those with lower hippocampal reserve. Although LPA did not mediate the sleep-memory relationship, it remained an independent modifiable factor, also moderated by brain reserve. Together, sleep continuity and light physical activity represent key behavioral targets for cognitive preservation in aging.

## Discussion

### Principal Findings

In this study, using wearable device–based monitoring, we identified 2 objectively measured lifestyle factors—LPA time and WASO—that were significantly associated with memory performance in community-dwelling older adults. Higher LPA time was linked to better memory scores, while greater WASO was associated with poor memory. These associations remained robust after adjusting for age, education, daytime sleepiness, and hippocampal volume. Importantly, age moderated both effects, with LPA showing a protective role from age 73.8 years onward, and WASO exerting a detrimental influence from age 71.1 years onward. Subgroup regression analyses confirmed that both LPA (β=.054; *P*=.04) and WASO (β=–0.111; *P*=.01) were stronger predictors of memory among older participants (≥72 years of age). Moreover, hippocampal volume moderated these associations, consistent with the brain reserve hypothesis.

Beyond these primary findings, this study contributes novel insights in 3 ways. First, it simultaneously examined sleep and physical activity using objective wearable-based monitoring, rather than focusing on one lifestyle factor in isolation. Second, it integrated these measures with validated neuropsychological testing and structural brain imaging, providing a multimodal framework to explore behavioral and neural determinants of cognitive health. Third, by testing age and hippocampal reserve as moderators, it highlights how the effects of lifestyle factors intensify with advancing age and reduced neural reserve. Taken together, this work advances understanding of how modifiable lifestyle behaviors interact with biological aging processes to influence memory in older adults, particularly within an underrepresented Asian population.

This study highlights LPA and sleep continuity (measured as WASO) as key modifiable lifestyle factors associated with memory performance in community-dwelling older adults. Our findings on the correlation between sleep and memory function align with those of other studies. Actigraphy-based research has identified links between sleep disturbances, prolonged sleep duration, and poorer cognitive performance [6,12,20,29]. Recent evidence from an accelerometer-based study in *JMIR Aging* further demonstrated that greater night-to-night variability in sleep efficiency was associated with poorer cognitive performance in older adults [30], reinforcing the role of objective sleep fragmentation in cognitive aging. Consistently, wearable-derived rhythm metrics, such as interdaily stability and sleep-wake fragmentation measured by the myRhythmWatch platform, have been linked to cognitive impairment among community-dwelling elders [31], supporting the concept that circadian rhythm disruption contributes to cognitive vulnerability. Similarly, good sleep quality, as objectively measured by polysomnography, has been shown to correlate with enhanced cognitive performance in older adults [11]. Various potential mechanisms have been proposed to explain how sleep influences cognition in older people. Sleep disturbances might negatively impact synaptic plasticity by disrupting neuronal and glial signaling pathways [32]. Inadequate sleep could promote neuroinflammation and impair neurogenesis, potentially leading to neurodegeneration in the hippocampus [33]. Furthermore, increased sleep fragmentation, often due to sleep-disordered breathing, could induce neuron apoptosis and brain atrophy through inflammatory and oxidative pathways [34]. A polysomnography-based study also highlighted the relationship between sleep and glymphatic function, which, in turn, was associated with neuropsychological function [35] and gray matter volumes [36].

Numerous studies have identified physical activity as a crucial, modifiable lifestyle factor influencing cognitive and memory function in aging populations [14,37]. However, the optimal duration, frequency, and intensity of physical activity necessary to mitigate cognitive decline in older adults remain uncertain. Several mechanisms have been proposed to explain the cognitive benefits of physical activity. One key hypothesis centers on its impact on brain structure; that is, MRI-based studies have shown that regular activity is associated with preserved brain microstructure and reduced hippocampal atrophy [38,39]. At the molecular level, exercise stimulates the release of myokines from skeletal muscle, promoting the production of neurotrophic factors such as brain-derived neurotrophic factor and insulin-like growth factor-1 [18]. These neurotrophic mediators support synaptic plasticity, neurogenesis, and angiogenesis in regions critical to memory, including the hippocampus [40-44]. This interaction between muscle and brain could account for the observed benefits of physical activity on cognitive function in older people. Reports suggest that higher intensity and variability in physical activity are linked to a reduced risk of dementia [45,46]. While MVPA is often the focus of public health recommendations, our findings indicate that even low-intensity movement, particularly LPA, plays a meaningful role in supporting cognitive health. Interestingly, in our relatively healthy older adult sample, 75% (66/88 participants) reported having regular exercise habits exceeding 150 minutes per week, and accelerometry data showed that MPA time was substantially higher than LPA time (mean MPA 236.2, SD 67.6 minutes/day vs mean LPA: 141.5, SD 33.2 minutes/day). The absence of a significant association between MPA and memory in our data may partly reflect a ceiling effect, as most participants already met or exceeded recommended activity levels. However, this explanation remains speculative and was not directly tested. Future studies with broader variability in physical activity levels are warranted to verify this interpretation. In contrast, LPA, which includes incidental movement such as light walking, standing, or household activities, showed greater interindividual variation and a stronger correlation with cognitive function. Consistent with this interpretation, supplementary analyses (Figure S1 in Multimedia Appendix 1) demonstrated that neither MPA, VPA, nor their combined MVPA time showed significant age-moderated associations with memory performance, further supporting that low-intensity, sustained movement may be a more sensitive behavioral indicator of cognitive health in highly active older adults.

Our findings extend the emerging evidence supporting incidental light physical activity as a health-promoting behavior. A recent large-scale study [47] demonstrated that incidental light physical activity was associated with a dose-dependent reduction in cardiovascular events and all-cause mortality, even in populations not engaging in structured exercise. Building on these results, our study provides novel evidence that LPA is also beneficial for brain health, particularly memory performance in older adults. Additionally, physical activity may contribute uniquely to cognition through nonfatiguing, sustained movement that stabilizes cerebral perfusion, minimizes sleep disruption, and potentially supports glymphatic clearance. Recent evidence suggests that long-term exercise can enhance brain waste removal via glymphatic and meningeal lymphatic pathways [48], providing a novel mechanism linking physical activity with cognitive health. Taken together, these findings suggest that while maintaining structured exercise remains important [49], encouraging older adults to stay consistently active throughout the day, even at low intensity, may be an equally vital strategy for preserving memory and cognitive function.

Our findings indicate that age significantly moderates the impact of lifestyle factors on memory performance. Using Johnson-Neyman analysis, we identified distinct age thresholds—73.8 years for LPA and 71.1 years for WASO—beyond which the cognitive effects of these modifiable behaviors became statistically significant. While both LPA and WASO were significant predictors of memory performance in the overall sample, subgroup regression analyses showed that their predictive effects were even stronger among participants aged ≥72 years. This observation aligns with emerging evidence that aging is not a uniform or linear process but one marked by biological inflection points across the lifespan. For instance, a large-scale proteomic study [50] revealed 3 major age-related transitions in the human plasma proteome—around ages 34, 60, and 78 years—indicating phase-like shifts in biological aging trajectories. Similarly, a recent multiomics analysis [51] showed cliff-like drops in functional integrity occurring around ages 44 and 60 years, supporting a model of nonlinear, stepwise aging rather than gradual decline. While our study does not directly assess molecular aging, our results extend this nonlinear perspective by showing that the cognitive impact of behavioral factors such as physical activity and sleep continuity intensifies with advancing age. These findings carry important clinical implications. They suggest that behavioral risks are not static across age but rather become more influential in late life, even in otherwise healthy older adults. This emphasizes the importance of early and consistent engagement in health-promoting behaviors throughout adulthood, while also recognizing that targeted interventions in older age may yield greater benefits. Age-stratified strategies that prioritize sleep consolidation and daily activity may help optimize cognitive preservation throughout aging.

Our exploratory analyses suggest that the relationship between sleep and memory is not only behavioral but also shaped by neurobiological and age-related factors. Although sleep fragmentation (WASO) was associated with reduced LPA in older adults, this behavioral pathway did not significantly mediate memory outcomes. Instead, WASO exerted a robust and direct negative influence on memory, with the effect strengthening significantly with increasing age. The absence of a significant indirect pathway suggests that sleep fragmentation may impair memory primarily through direct neurophysiological mechanisms—such as disrupted synaptic homeostasis or impaired glymphatic clearance—rather than solely through behavioral reductions in activity. This interpretation aligns with prior evidence linking sleep continuity to neural restoration and waste clearance processes. In parallel, hippocampal volume moderated the associations of both WASO and LPA with memory performance. Participants with larger hippocampi showed relative resilience to the adverse effects of poor sleep and low activity, whereas those with smaller hippocampal volume demonstrated stronger behavior-cognition associations. These findings support the brain reserve hypothesis, which posits that individuals with greater structural brain integrity are more capable of withstanding behavioral and pathological insults without manifesting cognitive decline. While hippocampal volume has previously been linked to cognitive resilience in the context of neuropathology, depression, and sleep disorders [52-54], our results extend this model to modifiable lifestyle factors measured objectively via wearable technologies in cognitively healthy older adults.

To explore the reasons behind the varying correlation patterns between sleep, physical activity, and memory function in participants older than 72 years, we compared several physical, laboratory, and imaging parameters between participants younger and older than this age threshold. Significant differences were observed in some variables, such as pulse rate, visceral fat rating, the ratio of ECW to TBW, indicators of muscle function, and hippocampal volume, but not in the appendicular skeletal muscle mass index between the 2 groups. Previous studies have indicated that higher levels of body fat negatively affect brain volume and contribute to memory decline [55]. Furthermore, higher ratios of ECW to TBW have been linked to poorer cognition [56]. Whether these differences in body composition are responsible for diminishing the effects of physical activity on memory function in individuals aged 72 years or older requires further investigation.

In addition to these physiological factors, systemic inflammation may also play a role in mediating age-related differences. Inflammatory markers, including interleukin-1β, interleukin-6, interleukin-10, and tumor necrosis factor-alpha, were assessed in this study (Table S1 in Multimedia Appendix 1). Among these, only interleukin-8 levels were significantly higher in older adults, while other cytokines showed no marked differences. This pattern suggests that low-grade inflammation may exist but remains relatively mild in this healthy, community-dwelling sample. The modest change in IL-8 could reflect early immune activation associated with aging and sleep disruption, consistent with prior evidence linking chronic sleep fragmentation to subtle increases in inflammatory signaling [57-59]. Future studies incorporating oxidative stress and glial activation markers could help clarify whether inflammatory processes mediate the observed age-specific behavioral and cognitive effects.

In this study, we used the Actiwatch 2 accelerometer to objectively assess physical activity and estimate sleep quality and duration. As a lightweight and easy-to-wear device, actigraphy enables continuous monitoring over multiple days or weeks [60], thereby capturing behavioral patterns that more closely reflect individuals’ habitual, real-world lifestyles. While actigraphy is known to overestimate TST and underestimate nocturnal awakenings when compared to polysomnography [60], it remains a practical and scalable tool in population-based and longitudinal research [61]. In an era of increasingly accessible wearable technology, actigraphy offers valuable insight into daily lifestyle rhythms and provides meaningful, ecologically valid data to inform behavioral recommendations and health interventions, especially in community and aging populations where in-laboratory polysomnography is often not feasible.

Our findings contribute new insights by integrating wearable-derived behavioral data, validated neuropsychological memory outcomes, and structural brain imaging into a unified analytic model. The demonstration that sleep and activity behaviors interact with age and hippocampal reserve to influence memory performance underscores the complexity and heterogeneity of cognitive aging. Importantly, these results suggest that individuals with lower hippocampal volume—who may be at higher risk of future cognitive decline—could particularly benefit from targeted interventions that improve sleep continuity and promote consistent physical activity. This highlights the potential value of combining lifestyle-based strategies with brain-based risk stratification in the design of personalized cognitive health programs for older adults.

From a translational perspective, these findings underscore the potential of promoting incidental, low-intensity movement, such as walking, household activities, and standing breaks, as realistic behavioral goals for older adults who may find structured exercise challenging. Public health strategies emphasizing total daily movement, rather than only moderate to vigorous exercise, may therefore be particularly beneficial in aging populations.

This study has several notable strengths. First, we used objective, wearable-based monitoring to assess both sleep and physical activity over an extended period in a real-world setting, avoiding the biases associated with self-reported lifestyle data. Second, by focusing on memory-specific cognitive outcomes, rather than global cognition, we provide a more targeted understanding of functional decline in aging. Third, we incorporated structural neuroimaging data to test how brain reserve, operationalized as hippocampal volume, interacts with behavioral risk factors. To our knowledge, this is one of the few studies to examine the joint impact of objectively measured lifestyle factors and neural reserve on memory performance in healthy older adults, using advanced moderation and mediation models to capture complex interactions between age, behavior, and brain structure.

Despite these strengths, several limitations should be acknowledged. First, although this was a prospectively designed cross-sectional study, the absence of longitudinal follow-up limits our ability to infer causal relationships between physical activity, sleep, and memory function. Future longitudinal or interventional studies are warranted to verify the temporal and causal pathways suggested by our findings. Second, participants were recruited from a single hospital-based health examination program in northern Taiwan, which may introduce selection bias and limit external validity. Third, our sample consisted primarily of relatively healthy, community-dwelling older adults with generally high physical activity levels. While this ensured sample homogeneity and reduced confounding, it also excluded high-risk individuals, such as those with cognitive impairment or major chronic diseases, thus limiting the generalizability of our results to more clinically vulnerable populations. Fourth, participants were aware that their activity and sleep were being monitored, which may have temporarily increased activity levels and slightly reduced the accuracy of habitual behavior estimates. Fifth, although actigraphy is not the gold standard for evaluating sleep architecture or activity intensity, it remains a US Food and Drug Administration–approved, user-friendly tool that enables unobtrusive and ecologically valid data collection under free-living conditions. In an era of widespread wearable technology use [62], actigraphy offers scalable and meaningful behavioral data for aging research. Sixth, polysomnography data were collected as part of the broader ISDHA cohort but were not included in this analysis because this study focused on wearable-based lifestyle measures to capture real-world behavior; polysomnography findings will be reported separately. Seventh, we did not account for other potentially influential factors, such as diet, socioeconomic status, or social engagement, that may also modulate cognitive outcomes. Finally, although hippocampal volume was examined as a moderator, other neural or molecular mechanisms (eg, functional connectivity or serum neurotrophic and inflammatory markers) were not analyzed. While basic inflammatory cytokines were assessed, more specific biomarkers of oxidative stress or neuroinflammation (eg, glial or endothelial activation indices) should be incorporated in future studies to clarify underlying biological pathways. Additionally, the proposed ceiling effect for moderate physical activity should be interpreted cautiously, as it was not formally tested. Collectively, these limitations highlight the need for longitudinal, multisite studies integrating multimodal biomarkers to validate and expand our findings. Nonetheless, this study provides an essential foundation for identifying modifiable behavioral factors and developing age- and brain-based strategies to preserve cognitive health in older adults.

### Conclusions

In this study of cognitively healthy older adults, we demonstrated that 2 modifiable lifestyle factors—sleep continuity and LPA, objectively measured via wearable devices—were significantly associated with memory performance. These associations were further shaped by age and hippocampal volume, emphasizing the interplay between behavioral and neurobiological factors in cognitive aging. Our findings provide empirical support for the brain reserve hypothesis and highlight that the cognitive impact of lifestyle behaviors becomes more pronounced in later life and among individuals with lower neural reserve. Encouraging consistent, light-intensity activity throughout the day, together with maintaining sleep continuity, may represent a practical, scalable, and age-tailored strategy to support cognitive health in older adults. Future longitudinal and intervention studies are warranted to validate these pathways and inform personalized behavioral approaches for preserving memory function across aging populations.

## Appendix

Multimedia Appendix 1 Interactions of physical activity measures with age on memory performance, and age-group differences in physical, laboratory, and brain magnetic resonance imaging characteristics.

## Abbreviations

AD8: Ascertain Dementia 8
CERAD-NB: Consortium to Establish a Registry for Alzheimer’s Disease Neuropsychological Battery
ECW: extracellular water
ESS: Epworth Sleepiness Scale
GDS: Geriatric Depression Scale
ISDHA: Integrating Systematic Data of Geriatric Medicine to Explore the Solution for Healthy Aging
L5: mean activity counts per minute during the least active 5 consecutive hours
LPA: light-intensity physical activity
M10: mean activity counts per minute during the most active 10 consecutive hours
MET: metabolic equivalents of task
MMSE: Mini-Mental State Examination
MPA: moderate-intensity physical activity
MRI: magnetic resonance imaging
MVPA: moderate to vigorous physical activity
PSQI: Pittsburgh Sleep Quality Index
SB: sedentary behavior
TBW: total body water
TIB: time in bed
TST: total sleep time
VPA: vigorous-intensity physical activity
WASO: wake after sleep onset

## Data Availability

The data collected and analyzed for this study are available from the corresponding author on reasonable request.
