# Supplementary material for: Associations of Wearable-Measured Sleep and Physical Activity With Memory Performance in Older Adults: Cross-Sectional Study With Actigraphy and MRI
Source: JMIR Aging. 2025 Dec 9;8:e80584. doi: 10.2196/80584 (PMC12728405; doi:10.2196/80584)
Supplement: Multimedia Appendix 1 [file aging_v8i1e80584_app1.docx]

**Figure S1.** Interactions of moderate-intensity physical activity (MPA), vigorous-intensity physical activity (VPA), and combined moderate-to-vigorous physical activity (MVPA) time with age on memory performance.


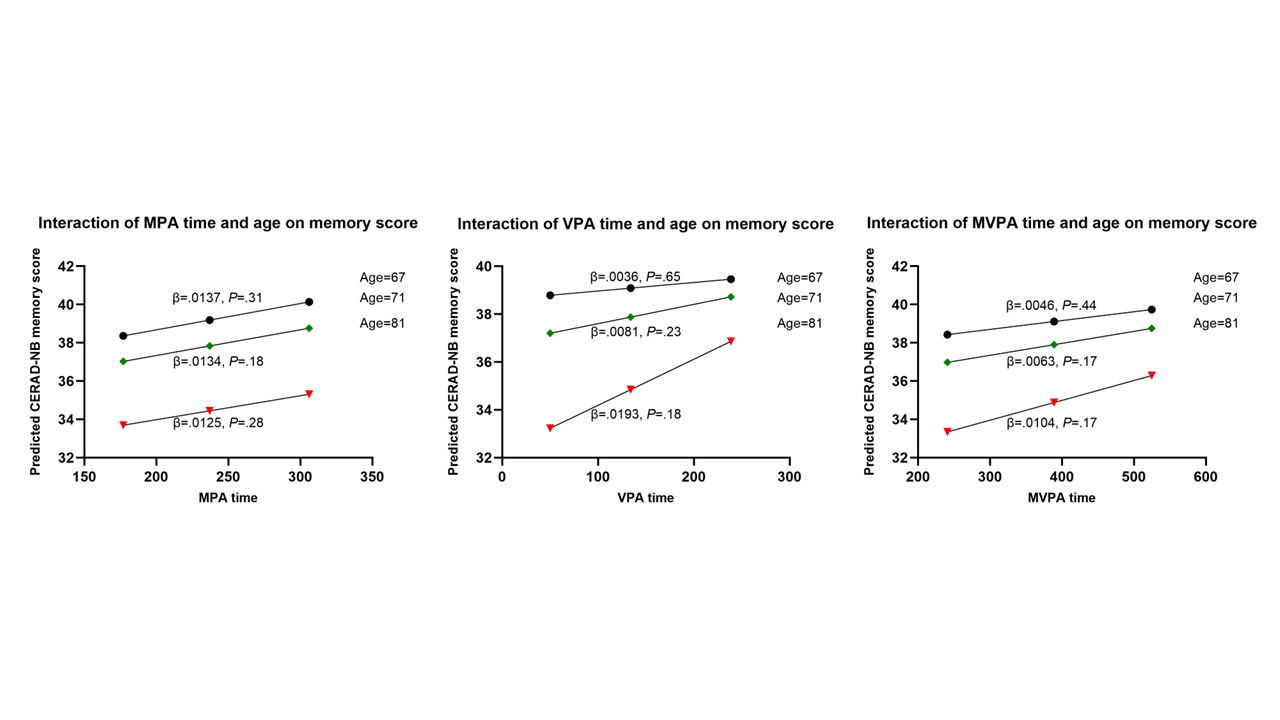


Predicted CERAD-NB memory scores are plotted as a function of physical activity time at representative ages (67, 71, and 81 years). No significant age-moderated effects were observed for MPA, VPA, or MVPA on memory performance.

**Table S1.** Group differences in physical, laboratory, and brain MRI measures between participants aged <72 and ≥72 Years

| Variables (mean±SD) | Age | | *P* value |
| --- | --- | --- | --- |
|  | <72 | ≧72 |  |
| Vital Signs |  |  |  |
| Systolic Blood Pressure | 135.0±14.7 | 141.7±18.5 | .08 |
| Diastolic Blood Pressure | 74.9±10.3 | 75.1±8.8 | .95 |
| Pulse rate | 75.5±9.0 | 69.9±11.1 | .012 |
| Respiratory rate | 15.4±2.3 | 15.7±1.5 | .553 |
| Anthropometry and BIA Measures |  |  |  |
| Height | 161.7±8.0 | 160.3±6.7 | .528 |
| Weight | 61.0±11.0 | 62.3±10.7 | .667 |
| BMI | 23.2±2.8 | 24.2±3.8 | .325 |
| Fat mass | 16.0±5.0 | 17.9±8.1 | .367 |
| Fat | 26.1±6.8 | 28.2±8.6 | .253 |
| Fat free mass | 45.1±9.1 | 44.4±7.3 | .947 |
| Visceral Fat Rating | 10.0±4.1 | 12.2±4.5 | .020 |
| Muscle mass | 42.6±8.7 | 41.9±7.0 | .94 |
| Bone mass | 2.4±0.4 | 2.4±0.4 | .876 |
| Total body water (TBW) | 31.4±5.9 | 31.5±5.0 | .748 |
| Extracellular water (ECW) | 13.8±2.1 | 14.2±1.8 | .312 |
| Intracellular water (ICW) | 17.7±3.9 | 17.4±3.2 | .726 |
| ECW/TBW | 44.1±2.1 | 45.2±2.2 | .029 |
| Basal metabolic rate | 1259.9±232.0 | 1240.7±179.2 | .933 |
| Appendicular Skeletal Muscle Mass (ASM) Index | 6.8±1.2 | 6.9±1.3 | .99 |
| Physical Function Tests |  |  |  |
| Gait speed | 1.3±0.3 | 1.0±0.3 | <0.001 |
| Grip strength | 30.2±9.0 | 26.4±7.0 | .098 |
| Complete Blood Count (CBC) |  |  |  |
| WBC | 5.5±1.1 | 5.5±1.5 | .713 |
| RBC | 4.6±0.4 | 4.4±0.5 | .019 |
| Hemoglobin | 14.0±1.1 | 13.6±1.4 | .124 |
| Hematocrit | 41.8±3.0 | 40.5±4.1 | .133 |
| Platelets | 222.5±49.9 | 208.0±47.3 | .202 |
| Serum Metabolic Profile |  |  |  |
| Total cholesterol | 191.3±33.4 | 191.4±36.7 | .986 |
| Low-density lipoprotein cholesterol | 118.3±28.3 | 117.5±31.2 | .962 |
| High-density lipoprotein cholesterol | 57.7±14.8 | 57.2±15.9 | .892 |
| Triglyceride | 110.8±52.5 | 108.8±43.4 | .875 |
| Glucose AC | 95.3±13.6 | 99.1±27.2 | .625 |
| HbA1c | 5.9±0.4 | 6.0±0.7 | .365 |
| Uric acid | 5.4±1.4 | 5.7±1.4 | .327 |
| Serum Biochemistry (renal, hepatic, nutritional) |  |  |  |
| BUN | 15.7±3.8 | 18.8±6.1 | .015 |
| Creatinine | 0.8±0.2 | 0.9±0.3 | .017 |
| Estimated GFR | 91.1±22.4 | 73.6±22.1 | <0.001 |
| Ca | 9.1±0.4 | 9.0±0.3 | .693 |
| Inorganic P | 3.6±0.6 | 3.6±0.5 | .983 |
| AST | 27.1±7.9 | 26.5±9.6 | .301 |
| ALT | 25.7±11.2 | 20.5±9.0 | .001 |
| Alkaline P | 61.6±18.9 | 55.2±14.6 | .146 |
| rGT | 21.6±10.3 | 18.8±7.7 | .345 |
| Total protein | 7.1±0.5 | 7.1±0.4 | .957 |
| Albumin | 4.4±0.2 | 4.3±0.2 | .054 |
| Vitamin D | 27.8±8.2 | 29.8±9.1 | .434 |
| Inflammatory Markers |  |  |  |
| IL-1b | 33.8±111.9 | 52.2±202.2 | .89 |
| IL-6 | 2829.1±10676.4 | 36395.1±202178.7 | .451 |
| IL-8 | 1297.9±1203.5 | 2140.2±2007.6 | .018 |
| IL-10 | 406.5±290.6 | 430.4±245.3 | .32 |
| TNF-a | 318.5±834.5 | 119.0±406.3 | .511 |
| Structural Brain MRI Measures |  |  |  |
| Total volume | 1490.1±146.1 | 1482.7c±141.1 | .697 |
| Grey matter | 550.1±58.0 | 484.8±73.8 | <0.001 |
| White matter | 473.6±58.5 | 455.3±55.2 | .084 |
| White matter hyperintensity (WMH) | 10.4±13.3 | 26.3±35.2 | .036 |
| Right hippocampus | 3.2±0.3 | 2.8±0.3 | <0.001 |
| Left hippocampus | 2.8±0.3 | 2.4±0.3 | <0.001 |
| Right anterior cingulate cortex | 0.4±0.0 | 0.3±0.0 | <0.001 |
| Left anterior cingulate cortex | 0.4±0.0 | 0.3±0.1 | <0.001 |
